# Association among Weather Conditions, Ambient Air Temperature, and Sedentary Time in Chinese Adults

**DOI:** 10.1155/2019/4010898

**Published:** 2019-12-28

**Authors:** Xu Wen, Yiqun Ma, Bing Yuan, Fubaihui Wang

**Affiliations:** ^1^Department of Sport & Exercise Science, College of Education, Zhejiang University, Hangzhou 310028, China; ^2^Information Technology Center, Zhejiang University, Hangzhou 310028, China; ^3^China Institute of Sport Science, Beijing 100061, China

## Abstract

This study is aimed to quantify the association among weather conditions, ambient air temperature, and sedentary time in Chinese adults. The participants were 3,270 Chinese users of a wrist-worn activity tracker. Their daily activity data were collected using an algorithm based on raw data to determine the sedentary time. The data of ambient air temperature and weather were collected from the meteorological data released by China Central Meteorological Observatory. Two-level linear regression analyses showed that weather conditions had a significant influence on sedentary time in Chinese adults after adjustments for some covariates were made. When the weather condition changed from rainy days to sunny and cloudy days, sedentary time might decrease by about 6.89 and 5.60 min, respectively. In conclusion, weather conditions were independently associated with sedentary time in Chinese adults. The daily sedentary time was shorter on sunny and cloudy days than on rainy days.

## 1. Introduction

Sedentary behavior, which is defined as any waking behavior with a sitting, reclining, or lying posture that requires an energy expenditure of ≤1.5 metabolic equivalents [1], has emerged as a new research focus concerning physical activity and health. Several large population-based epidemiological studies have indicated that sedentary behavior, independent of physical activity, is associated with the development of several chronic diseases, including obesity, diabetes, and cardiovascular diseases [2, 3]. An increasing number of studies have found that some sedentary behaviors are associated with high depressive symptoms and poor cognitive function [4]. Furthermore, “breaking-up” sedentary duration may positively affect physical function and blood pressure [5]. These studies have suggested that decreasing sedentary time is an important strategy to maintain and improve health status.

Weather conditions and ambient air temperature, which significantly influence human health and well-being, have been investigated. Numerous epidemiological studies have suggested that extremes of temperature are associated with a short-term increase in mortality [6–8]. The effects of cold weather on cardiac mortality have also been demonstrated [9, 10]. Weather and temperature influence the psychological health of children and adults. Studies have indicated that suicide rates among the elderly may be affected by deviations of monthly mean temperature from values expected for that time of the year [11], whereas the suicide rates among adolescents are associated with season [12]. A study on weather and subjective well-being has indicated that self-reported life satisfaction decreases with the amount of rain on the day of an interview [13]. Low temperatures increase happiness and reduce tiredness and stress, raising the net effect; by contrast, high temperatures reduce happiness [13].

Although the dose-response effects of weather and air temperature on physical activities have been described [14–16], limited studies have been conducted on the association between weather and sedentary behavior [17–19]. A study based on 480 children in the United Kingdom has shown that the time spent in sedentary behavior is longer in spring, autumn, and winter compared with that spent in summer [18]. However, a cross-sectional study with 1,172 participants in Sweden has revealed no significant effects of season on time spent in sedentary behavior [19]. A probable reason for these inconsistent results is that seasonal variation in sedentary behavior can be location specific because of regional differences in climate [17]. In a recent study involving the use of an accelerometer, high precipitation and low temperatures are associated with 15 min more sedentary time in comparison with long day length and good weather conditions [20]. In summary, findings on weather conditions, ambient air temperature, and sedentary behavior are still inconclusive because of the incomparable definition of the season [17], shortage of related studies, especially in adults, and lack of the objectively measured data of sedentary time. Therefore, this study is aimed to quantify the association among weather conditions, ambient air temperature, and sedentary time in adults by utilizing an activity tracker with a novel algorithm to measure sedentary time.

## 2. Methods

### 2.1. Participants

The participants were 4,604 Chinese users of Bong II, a popular brand of a wrist-worn activity tracker in China. This brand of tracker has thousands of users. Approximately 20,000 users were selected randomly, and they were contacted via email with the assistance of Hangzhou Gongke Technology Co., Ltd. In this email, we introduced the purpose and content of the research and invited them to participate and provide their data. Only users who agreed to participate were recruited in this study. The study was approved by the Ethics Committee of the China Institute of Sport Science.

The data of the participants' daily activities were collected from July to October 2015 in 33 of 34 provinces, autonomous regions, and municipalities in China. The inclusion standards for data analysis were set as follows: (1) aged 18 or above, (2) without disability and critical diseases, (3) wear time ≥18 h per day and ≥4 consecutive days, (4) available global positioning system (GPS) data, and (5) available meteorological data. The data of 3,270 participants (2,021 men and 1,249 women) were included in the analysis.

### 2.2. Data Collection

Sedentary time was measured using a Bong II activity tracker. This tracker was 14 g in weight, 4 cm in length, 2 cm in width, and 0.9 cm in thickness. It had an adjustable wristband and was required to be worn in the nondominant wrist to avoid influencing the participants' normal activities. The product was waterproof and could record sleeping, which made 24 h of wearing possible. This tracker had a three-axis accelerometer in the activity monitor. The sampling frequency was 100 Hz, and the output data rate was 1–15 s epoch based on the type of activities. The raw 100 Hz files were converted using Bong 2.0 to 15 s epoch files containing *x*, *y*, and *z* vectors (mean acceleration over the epoch, retaining the gravity vector) and vector magnitude values (summed over the epoch, corrected for gravity).

The algorithm applied to the current study was Sedentary Sphere, which is based on the collected raw data of the accelerometer and gravity component of the acceleration signal to classify the mode of activities (e.g., sitting/lying, standing, walking, jogging, and sleeping) [21, 22]. The method was introduced in detail in previous studies and found to be valid and reliable in measuring sedentary behavior [21, 22].

The posture identifications followed a principle based on arm elevation. The arm was elevated if the data plotted on the Sedentary Sphere in latitudes at elevation were greater than 15° below the horizontal, possibly indicating a sitting or reclining posture if a low physical activity (determined by the vector magnitude of acceleration signal) was also found. A standing position was suggested if the data plotted were lower than 15° below the horizontal. The validity of wrist-worn accelerometers with Sedentary Sphere measuring sedentary time in free-living adults by using activPAL as the “gold standard” was reported in several studies. The sitting time measured with activPAL and a wrist-worn tracker with Sedentary Sphere was reported to be significantly correlated (rho = 0.9, *p* < 0.001) [21]. The intraclass correlation coefficient (ICC) between the sedentary time measured with a tracker with Sedentary Sphere and activPAL was found to be 0.93 (95% confidence interval 0.84–0.97) [22]. The mean intraindividual agreement of this measurement was reported to be 85% ± 7% [21].

In the preliminary research on the reliability and validity of the activity tracker in the current study, 32 undergraduate students (18 men and 14 women) were instructed to wear the Bong II activity tracker on their nondominant wrist and activPAL on their right thigh for 7 consecutive days. activPAL is a small lightweight triaxial accelerometer, which has been demonstrated to be a valid tool for identifying sedentary behaviors [23, 24]. The accuracy of the monitor in classifying lying, sitting, and upright activities was 89%. The ICC between the sedentary time measured with the Bong II activity tracker and activPAL was 0.78 (95% confidence interval = 0.68–0.86). The Bland–Altman plot of the data indicated that only 8.9% of the data were outside the mean ± 1.96 standard deviations. Using activPAL as the “gold standard”, the mean difference was 18 min and the limits of agreement were −76 min and +82 min.

Personal information, including age, sex, height, weight, and physical condition, was self-reported on the App of Bong II by the participants when they first used the product. Regions of residence were obtained via GPS. The data of ambient air temperatures, weather, and air quality index (AQI) were collected from the meteorological data released by China Central Meteorological Observatory by using GPS and time information. AQI was divided into four categories as follows: excellent (AQI = 0–50), good (AQI = 50–100), polluted (AQI > 100), and moderately and heavily polluted (AQI > 150). Temperature was categorized into four groups (Q1, Q2, Q3, and Q4) by using quartiles as cut-off points. Wake time was determined on the basis of the data of accelerometers by using the algorithm applied in previous studies [25–27].

### 2.3. Data Analysis

Only the records without missing the data of sedentary time, GPS, air quality, weather conditions, and temperature were included in the final analysis. Two-level linear regression analyses were conducted to investigate the association among weather conditions, ambient air temperature, and sedentary time. The records of each participant on each day were nested within individuals. The association among weather conditions, ambient air temperature, and sedentary behavior between days was anticipated to exhibit temporal autocorrelation, so the repeated covariance type was set as the first-order autoregressive structure. After a null model was used to determine whether a multilevel analysis should be applied, the unadjusted and adjusted associations among sedentary time, individual factors (age, gender, BMI, and region), and other potential covariates (weekday/weekend classification and air quality) were examined. Unadjusted and adjusted associations among weather conditions, ambient air temperature, and sedentary time were investigated. All statistical analyses with a significance level of *p*=0.05 were conducted using IBM SPSS 20.0.

## 3. Results

The demographic information of the participants is presented in Table 1. The range of age was from 18 years to 86 years. The mean monitored duration was 11.5 days in men and 11.3 days in women, and they were longer than the monitored time in most previous studies [28–30]. Person-day is the sum of the numbers of the monitored days of all participants, and a total of 37,361 person-days were recorded.

The associations between sedentary time and potential covariates are shown in Table 2. The results show that sex, weekday/weekend, and air quality were significantly associated with sedentary time. The men spent approximately 20 min more sedentary time than women did. People spent about 50 min more sedentary time on weekdays than on weekends. Sedentary time was longer on moderately and heavily polluted days than on days with better air quality.

Table 3 presents the weather condition and distribution of ambient air temperature. Cloudy and overcast were the most common weather conditions on the monitored days. Temperature was categorized into four groups (Q1, Q2, Q3, and Q4) by quartiles. The mean temperature was 22.1°C, with a range of −2°C to 40°C.

Multilevel analyses showed that weather conditions significantly influenced the sedentary time of Chinese adults even after adjustments for sex, air quality, weekday/weekend, and temperature were made (Table 4). When the weather condition changed from rainy days to sunny and cloudy days, sedentary time might decrease by about 6.89 and 5.60 min in Chinese adults. In the unadjusted model, ambient air temperature was associated with sedentary time. However, the association between temperature and sedentary behavior was not significant when adjustments for sex, air quality, weekday/weekend, and weather were made.

## 4. Discussion

This study quantified the association among weather conditions, ambient air temperature, and sedentary time in Chinese adults by using the data of the objectively measured sedentary time.

Our data suggested that weather conditions were associated with sedentary time in Chinese adults. The daily sedentary time was shorter on sunny days than on rainy days among Chinese men and women. This result was consistent with that of Eisinga et al. [31], who reported that uncomfortable weather conditions are associated with long daily television time.

This study indicated that sedentary time was significantly associated with ambient air temperature. However, when adjustments for some variables, including sex, air quality, weekday/weekend, and weather, were made, sedentary time was significantly associated with ambient air temperature. This finding was inconsistent with previous findings. Eisinga et al. [31] also reported that daily television time can be long on days with low temperatures. Other studies have also revealed that sedentary time is long in winter and short in summer [18, 32, 33]. Nevertheless, no significant difference in sedentary time is observed in different seasons in Sweden [19]. The seasonal variation in sedentary time can be location specific [17]. Our result could be possibly explained by the modification effect of air pollution. Air temperature influences the patterns of air quality over multiple scales in time and space through changes in air pollution emissions, transport, dilution, chemical transformation, and eventual deposition of air pollutants [34]. Hence, air quality is likely associated with sedentary time [35]. This internal relationship might help explain why air temperature became nonsignificant after the adjustment.

A possible explanation for the influence of weather and temperature on sedentary behaviors is that uncomfortable weather conditions and temperature decrease outdoor leisure activities but increase the probability of sedentary behavior [36]. Mood management theory may also help explain this phenomenon. Weather and temperature may influence the mood of people. For instance, sufficient sunshine, low humidity, and comfortable temperature are associated with high mood, whereas windy, cold, and dark days likely have a negative influence on mood [37]. By contrast, watching specific television programs, such as amusement and comedy, may help people improve their mood, thereby increasing sedentary time on days with uncomfortable weather and temperature [38]. In the current study, the data were consistent with mood management theory; that is, sedentary time on sunny days in Chinese adults was significantly longer than that on rainy days. Therefore, the mood may be an important mediator in the relationship between sedentary behaviors and weather or temperature. Future studies should be conducted to verify mood management theory in research on the physical environment and sedentary behavior.

Several limitations should be acknowledged when the results of this study would be interpreted. Firstly, the participants who were users of a particular brand of an activity tracker might share some common characteristics, such as socioeconomic status and exercise habits, such that they are not entirely representative of all Chinese citizens. Secondly, theoretically, only outdoor activities could be influenced by temperature and weather. However, the method and instruments used in the current study could not distinguish outdoor and indoor activities. Thirdly, sedentary time could differ with diurnal weather variations, but diurnal variations in weather and temperature were not analyzed in our study because hourly weather data in different regions were unavailable to support the analysis.

The strengths of this study included our relatively large sample size, objectively measured behavioral data, and application of a novel posture determination algorithm. In contrast to most previous studies that performed subjective methods, such as a questionnaire, this study utilized an activity tracker to measure sedentary time. Furthermore, instead of determining sedentary time merely based on energy expenditure, we applied Sedentary Sphere, a novel posture determination algorithm, to assist in the identification of sedentary behavior. Additionally, the utilization of an affordable commercial activity tracker allowed us to have a relatively large sample size in this study.

In summary, weather conditions were independently associated with sedentary time in Chinese adults. The daily sedentary time was shorter on sunny and cloudy days than on rainy days. When other environmental and individual factors were adjusted, no significant association between ambient air temperature and sedentary time was found.

## Figures and Tables

**Table 1:** Demographic information of participants.

	Total	Men	Women	*t*	*p*
Number of participants (%)	3270 (100)	2021 (61.8)	1249 (38.2)	—	—
Person-day	37361	23225	14136	—	—
Wearing time/day, hour; mean (SD)	22.69 (0.98)	22.67 (1.00)	22.71 (0.94)	−4.69^*∗∗∗*^	0.000
Monitored duration, day; mean (SD)	11.43 (4.15)	11.49 (4.11)	11.32 (4.20)	1.17	0.244
Age, year; mean (SD)	30.54 (7.44)	30.55 (7.52)	30.53 (7.32)	−0.08	0.933
Height, cm; mean(SD)	169.17 (7.98)	173.34 (6.51)	162.42 (4.93)	−54.31^*∗∗∗*^	0.000
Weight, kg; mean(SD)	59.39 (9.72)	63.66 (8.79)	52.49 (6.69)	−41.06^*∗∗∗*^	0.000
BMI, kg/cm^2^; mean (SD)	20.71 (2.76)	21.22 (2.89)	19.89 (2.32)	−14.50^*∗∗∗*^	0.000
Sedentary time/day, minute; mean (SD)	576.89 (146.93)	585.56 (149.22)	562.65 (141.94)	14.84^*∗∗∗*^	0.000

*Note.* ^*∗∗∗*^ *p* < 0.001.

**Table 2:** Associations between sedentary time (minutes) and potential covariates.

	Unadjusted			Adjusted^1^		
	Estimate (SE)	95% CI	*p*	Estimate (SE)	95% CI	*p*
Age						
18–29	12.51 (9.88)	−6.86, 31.89	0.205	13.41 (9.84)	−5.89, 32.70	0.173
30–39	4.04 (10.00)	−15.57, 23.64	0.686	5.16 (9.93)	−14.31, 24.64	0.603
40–49	−6.73 (11.27)	−28.83, 15.37	0.550	−4.42 (11.19)	−26.36, 17.52	0.693
≥50 (ref)	—		—	—	—	—
Gender						
Male	21.90 (3.29)^*∗∗∗*^	15.45, 28.35	0.000	22.53 (3.33)^*∗∗∗*^	16.00, 29.06	0.000
Female (ref)	—		—	—	—	—
BMI						
Underweight	−13.36 (28.67)	−69.57, 42.85	0.641	−26.31, 28.39	−81.96, 29.35	0.354
Normal	−12.95 (28.54)	−68.92, 43.00	0.650	−28.27, 28.27	−83.72, 27.16	0.317
Overweight	−11.19 (28.93)	−67.91, 45.53	0.699	−26.87, 28.68	−83.11, 29.37	0.349
Obese (ref)	—	—	—	—	—	—
Region						
North	−5.77 (3.50)	−12.63, 1.10	1.00	−5.27 (3.49)	−12.12, 1.58	0.132
South (ref)	—	—	—	—	—	—
Weekday/weekend						
Weekdays	48.57 (1.44)^*∗∗∗*^	45.74, 51.39	0.000	48.55 (1.45)^*∗∗∗*^	45.71, 51.39	0.000
Weekends (ref)	—	—	—	—	—	—
Air quality						
Excellent	−12.21 (6.56)	−25.08, 0.65	0.063	−9.85 (6.47)	−22.52, 2.83	0.128
Good	−17.44 (6.51)^*∗∗*^	−30.20, −4.69	0.007	−14.41 (6.40)^*∗*^	−26.96, −1.86	0.024
Lightly polluted	−9.10 (6.48)	−21.79, 3.60	0.160	−12.68 (6.37)^*∗*^	−25.17, −0.19	0.047
Moderately and heavily polluted (ref)	—	—	—	—	—	—

^1^The adjusted model included all six variables. ^*∗*^*p* < 0.05; ^*∗∗*^*p* < 0.01; ^*∗∗∗*^*p* < 0.001.

**Table 3:** Descriptive statistics of weather conditions and ambient air temperature.

	Days (%)
Weather	
Sunny	6703 (17.9)
Cloudy and overcast	12636 (33.8)
Rain	8703 (23.3)

Temperature	
Q1 (<20°C)	8038 (21.5)
Q2 (20–22.1°C)	9944 (26.6)
Q3 (22.1–25°C)	9993 (26.7)
Q4 (≥25°C)	9386 (25.1)

**Table 4:** Associations among weather conditions, ambient air temperature, and sedentary time (minutes).

	Unadjusted			Adjusted^1^		
	Estimate (SE)	95% CI	*p*	Estimate (SE)	95% CI	*p*
Weather						
Sunny	−8.04 (2.30)^*∗∗∗*^	−12.55, −3.52	0.000	−6.89 (2.32)^*∗∗*^	−11.45, −2.34	0.003
Cloudy and overcast	−3.67 (1.89)	−7.39, 0.05	0.053	−5.60 (1.90)^*∗∗*^	−9.33, −1.86	0.003
Rain (ref)	—	—	—	—	—	—

Temperature						
Q1 (<20°C)	2.95 (3.18)	−3.27, 9.18	0.353	−1.49 (3.38)	−8.13, 5.14	0.659
Q2 (20–22.1°C)	10.51 (2.76)^*∗∗∗*^	5.09, 15.92	0.000	5.09 (2.98)	−0.75, 10.95	0.088
Q3 (22.1–25°C)	6.12 (2.51)^*∗*^	1.20, 11.05	0.015	3.55 (2.72)	−1.80, 8.89	0.193
Q4 (≥25°C) (ref)	—	—	—	—	—	—

^1^Adjusted for gender, air quality, weekday/weekend, weather or temperature, and monitored wake time per day. ^*∗*^*p* < 0.05; ^*∗∗*^*p* < 0.01; ^*∗∗∗*^*p* < 0.001.

## Data Availability

The data used to support the findings of this study are available from the corresponding author upon request.
